# Pregnancy-Induced Exacerbation of Hereditary Angioedema in a Multiparous Caucasian Female

**DOI:** 10.7759/cureus.8006

**Published:** 2020-05-07

**Authors:** Praveen Sankrithi, Kunal Shah, Celina C Bernabe

**Affiliations:** 1 College of Osteopathic Medicine, Kansas City University of Medicine and Biosciences, Kansas City, USA; 2 Allergy and Immunology, Kansas University Medical Center, Overland Park, USA; 3 Allergy and Immunology, Menorah Medical Center, Overland Park, USA

**Keywords:** hereditary angioedema, pregnancy

## Abstract

Hereditary angioedema (HAE) manifests due to a deficiency of the C1-esterase inhibitor and can present with life-threatening swelling of multiple body regions such as the face, hands, upper respiratory tract, and intestinal walls. The present case describes the manifestation and symptomatic exacerbation of HAE in a multiparous Caucasian female. Very few trials and cases are available on HAE exacerbations during pregnancy, and our case describes the timeline and treatment in order to add to the clinical awareness of the disease. It is necessary to treat these patients rapidly to avoid unnecessary morbidity and interventions. For the time being, our patient has been appropriately managed with icatibant.

## Introduction

Hereditary angioedema (HAE) is a rare genetic condition. This disease manifests due to a deficiency of the C1-esterase inhibitor and presents with recurring episodes of swelling that can affect the face, hands, feet, lungs, and intestinal walls. C1-esterase inhibitor is a serine protease inhibitor that serves various functions in the fibrinolysis, complement, and contact systems. C1-esterase inhibitor prevents complement fixation and contacts plasma cascades to reduce bradykinin levels [1-7]. High levels of bradykinin cause vasodilation and increase vascular permeability, drawing vascular fluid into the subcutaneous space, leading to angioedema. These episodes can be life-threatening and potentially fatal. A rapid and accurate diagnosis is required because patients do not respond to the typical medications prescribed for the more common histamine-mediated angioedema [6,7]. However, there is limited information regarding the diagnosis and management of HAE in pregnant women. In this report, we describe a novel case of a patient with a history of HAE who experienced significant exacerbations of her condition during multiple pregnancies.

## Case presentation

A 35-year-old female with a history of HAE presented to the allergy clinic after being referred by her primary care physician. The patient was diagnosed with HAE when she was 17 years old and had a family history of HAE, with her mom and sister both being affected. When first diagnosed, the patient had suffered trauma to her foot that had resulted in excessive swelling for which she sought medical attention. Since then, she experienced acral and facial involvement but no laryngeal involvement. 

At 23 years of age, the patient became pregnant and her symptoms resurfaced. Her main complaint, during pregnancy, was abdominal pain. No medications were given during the pregnancy and her delivery (caesarian) was uncomplicated.

During her second pregnancy, at five months, she had experienced abdominal pain every two weeks for an eight-week time period. She was hospitalized for her symptoms and was given kalbitor to treat her attacks. The medication relieved her abdominal pain, and her delivery (cesarean) was without complications. 

Additionally, she became pregnant again a couple of years later and during the pregnancy, she had abdominal pain with emesis and evacuation, which resolved with no intervention. This prompted her referral to the allergy clinic. Triggers for her HAE attacks appear to be stress, trauma, oral contraceptives, and estrogen. 

Physical examination was grossly unremarkable at the time of her presentation. A nasal smear was ordered, which showed moderate white blood cells loaded with eosinophils. Moreover, a complete blood count (CBC) with differential and urinalysis (UA) with microscopy/culture were ordered and showed values within normal limits. A C1q binding assay was ordered and came back negative. Table 1 details further workup for potential complement deficiencies and to confirm a decreased C1-esterase inhibitor as would be expected in someone with HAE.

**Table 1:** Complement profile Normal limits are provided adjacent to the quantitative value

Test	Result (mg/dL)
Complement C4, serum	3 (9-36)
Complement C2	2.4 (1.6-4.0)
C1-esterase inhibitor, serum	8 (21-30)
Complement C1q, quantitative	8.8 (11.8-24.4)

The patient was started on 1000 units of cinryze (C1-esterase inhibitor [human]) therapy every four days and was told to follow up with her obstetrician/gynecologist (OB/GYN) for pain relief medications. Unfortunately, she developed adverse effects including localized edema described as “swelling in the abdomen.” The patient was changed to firazyr (icatibant; bradykinin inhibitor blocking the binding of bradykinin to the bradykinin B2 receptor) as needed for acute attacks.

## Discussion

HAE exacerbations during pregnancy, potential complications at delivery, and future implications are not well documented. Few studies have described HAE during pregnancy and potential treatments to improve outcomes and control symptoms. 

González-Quevedo et al., in 2016, described the management of pregnancy and delivery in patients with HAE. They used a retrospective review of 61 C1-esterase inhibitor deficient HAE patients. They measured the total number of pregnancies, changes in symptoms during delivery and pregnancy, type of anesthesia used, treatments, and tolerance of treatments. They reviewed 125 full-term pregnancies, 14 miscarriages, and four abortions and found that 59.2% of pregnancies (74/125) reported increased symptoms of HAE. They concluded that attacks tend to occur more frequently but did not increase in severity during pregnancy [3]. 

Machado et al., in 2017, have also described cases of pregnancy and postpartum care in HAE patients who have no access to therapy. Their findings confirmed that, in pregnancy, stress is the most common triggering factor followed by trauma. The extremities were the most frequently affected and most attacks occurred in the second trimester. They treated two patients with tranexamic acid and two patients with antihistamines. The difference between this report and our case is that this study took place in Brazil and the patients that were studied did not have access to specialized care during their pregnancy [5]. 

More information is needed about HAE in pregnancy in order to develop treatments. One avenue that is not well explored is genetic studies. Uncovering genetic markers that affect the severity of the disease and the effectiveness of treatments might lead to the prevention of attacks and reduction in symptom severity/frequency [2]. Chen and Riedl in 2018 describe new therapeutic innovations for the treatment of HAE including subcutaneous C1-esterase inhibitor concentrates, monoclonal antibody inhibitor of kallikrein, oral kallikrein inhibitors, ribonucleic acid (RNA) targeted antisense against pre-kallikrein, RNA interference drugs against factor XII, monoclonal antibody inhibitor of factor XIIa, and gene therapy. These advances in treatments could potentially be applied to patients in pregnancy, but more research should be conducted before medications like this are prescribed [1,6].

Additionally, further studies could be conducted on the role the complement system plays in pregnancy and HAE. An intact complement system optimizes placental development and is essential for host defense and fetal well-being. A number of pregnancy complications such as hypertensive disorders, preterm birth, and fetal growth restriction are associated with excessive or misdirected complement activation. Interestingly, Regal et al., in 2015, reported that the levels of C1-INH were decreased in 36-37 weeks of gestation in healthy women [8]. The role this plays in patients with HAE is unknown and is another area of potential future investigation for why the HAE symptoms are exacerbated during pregnancy.

Another potential mechanism for HAE exacerbation in pregnancy has to do with the role of estrogen. High levels of estrogen, which occurs during pregnancy, is associated with increased levels of factor XII. When activated, factor XII converts pre-kallikrein to kallikrein, which produces bradykinin from high molecular weight kininogen. Moreover, estrogen suppresses angiotensin-converting enzyme (ACE) resulting in reduced degradation of bradykinin [9]. These relationships offer a robust area for potential research towards the management and treatment of HAE in pregnant patients.

Ultimately, there are very few reports detailing the symptomatology and treatment of HAE exacerbations in multiparous Caucasian females, specifically. Preconception counseling, explanation of the disease, consultation to the specialized hospital, and close follow-up during pregnancy are very important to avoid severe complications in patients with HAE wishing for pregnancy. More research should be conducted to gain more information about potential mechanisms, effective treatments, and relationships between HAE and pregnancy.

## Conclusions

Our case represents a novel patient who had an exacerbation of her previously diagnosed HAE during her multiple gestations. Her variability in the presentation during each subsequent pregnancy is interesting and more information is needed to understand how multiparity, mode of delivery, and medications administered affect the outcomes of patients diagnosed with HAE. It is necessary to treat these patients quickly and, for the time being, icatibant has appropriately managed this patient’s symptoms.
